# Prognostic significance of fibroblast growth factor 19 (FGF19) expression in breast invasive ductal carcinoma

**DOI:** 10.1186/1471-2164-15-S2-P35

**Published:** 2014-04-02

**Authors:** Sahar Hakamy, Basmat Abdallah, Abdelbaset Buhmeida, Ashraf Dallol, Adnan Merdad, Jaudah Al-Maghrabi, Muhammad Abu-Elmagd, Mamdooh Gari, Adeel Chaudhary, Adel Abuzenadah, Taoufik Nedjadi, Eramah Ermiah, Fatima Thubaity, Mohammed Al-Qahtani

**Affiliations:** 1 Center of Excellence in Genomic Medicine Research, King Abdulaziz University, Jeddah, Kingdom of Saudi Arabia; 2 KACST Technology Innovation Center in Personalized Medicine, King Abdulaziz University, Jeddah, Kingdom of Saudi Arabia; 3 Department of Surgery, Faculty of Medicine, King Abdulaziz University, Jeddah, Kingdom of Saudi Arabia; 4 Department of Pathology, Faculty of Medicine, King Abdulaziz University, Jeddah, Kingdom of Saudi Arabia; 5 Faculty of Applied Medical Sciences, King Abdulaziz University, Jeddah, Kingdom of Saudi Arabia; 6 King Fahd Medical Research Center, King Abdulaziz University, Jeddah, Kingdom of Saudi Arabia; 7 Department of Oncology, National Cancer Institute, Sabratha, Libya

## Background

Several studies have shown that both FGF19 mRNA and protein are widely distributed in human tissues where they play an important role in cell proliferation, differentiation and motility (1-3). As part of our systematic search for prognostic markers in breast cancer (BC), the present study was conducted to assess the prognostic value of FGF19 in patients with BC.

## Materials and methods

Archival FFPE tumor samples were analyzed using immunohistochemistry (IHC) for monoclonal anti-FGF19 (W12) antibody in 193 patients with BC. IHC analysis was done using the automatic system (Bench-Mark XT; Ventana Medical Systems, Inc. Tucson, AZ, USA). Patients were diagnosed and treated at the Departments of Pathology, Surgery and Oncology, King Abdulaziz University Hospital, Saudi Arabia and the National Oncology Institute, Sabratha, Libya during years 2000-2008.

## Results

The expression pattern of FGF19 was predominantly cytoplasmic in the tumor area. Of the 193 tumors, 40% were considered low FGF19 expression, whereas 60% were considered high FGF19 expression. Interestingly, in lymph node positive patients, there was highly significant correlation between FGF19 expression and age of patients (p=0.008). Moreover, FGF19 expression showed significant correlation with tumor recurrence (p=0.02). Interestingly, in univariate (Kaplan-Meier) survival analysis, FGF19 expression was differentiating the DSS of lymph node positive tumors more significantly than the lymph node negative tumors (p<0.0001, log rank), in that tumors of lymph node positive patient with high FGF19 expression was more often, who eventually died of their disease (shorter disease specific survival (DSS)) as compared with those who were alive at the completion of the follow-up. On the other hand, PR status, tumor stage and grade had no significant relationship with FGF19 expression.

## Conclusions

Quantification of FGF19 expression seems to provide valuable prognostic information in BC, particularly in selecting lymph node positive patients who are at high risk for shorter DSS who might benefit from targeted therapy.

*This work was financially supported by King Abdulaziz City for Science and Technology* (*KACST*) *under research no* (*ASTP -10-Med-1107-03*)*.*
